# ‘Girls need to strengthen each other as a group’: experiences from a gender-sensitive stress management intervention by youth-friendly Swedish health services – a qualitative study

**DOI:** 10.1186/1471-2458-13-907

**Published:** 2013-10-01

**Authors:** Maria Strömbäck, Eva-Britt Malmgren-Olsson, Maria Wiklund

**Affiliations:** 1 Community Medicine and Rehabilitation, Physiotherapy, Umeå University, Umeå, Sweden; 2 Clinical Science, Psychiatry, Umeå University, Umeå, Sweden; 3 The National Graduate School for Gender Studies, Umeå Center for Gender Studies, Umeå University, Umeå, Sweden; 4 Umeå Center for Gender Studies, Umeå University, Umeå, Sweden

**Keywords:** Sweden, Young women, Adolescents, Youth mental health, Stress management, Intervention, Gender, Embodiment, Qualitative interviews, Physiotherapy

## Abstract

**Background:**

Mental health problems among young people, and girls and young women in particular, are a well-known health problem. Such gendered mental health patterns are also seen in conjunction with stress-related problems, such as anxiety and depression and psychosomatic complaints. Thus, intervention models tailored to the health care situation experienced by young women within a gendered and sociocultural context are needed. This qualitative study aims to illuminate young women’s experiences of participating in a body-based, gender-sensitive stress management group intervention by youth-friendly health services in northern Sweden.

**Methods:**

A physiotherapeutic body-based, health-promoting, gender-sensitive stress management intervention was created by youth-friendly Swedish health services. The stress management courses (n = 7) consisted of eight sessions, each lasting about two hours, and were led by the physiotherapist at the youth centre. The content in the intervention had a gender-sensitive approach, combining reflective discussions; short general lectures on, for example, stress and pressures related to body ideals; and physiotherapeutic methods, including body awareness and relaxation. Follow-up interviews were carried out with 32 young women (17–25 years of age) after they had completed the intervention. The data were analysed with qualitative content analysis.

**Results:**

The overall results of our interview analysis suggest that the stress management course we evaluated facilitated ‘a space for gendered and embodied empowerment in a hectic life’, implying that it both contributed to a sense of individual growth and allowed participants to unburden themselves of stress problems within a trustful and supportive context. Participants’ narrated experiences of ‘finding a social oasis to challenge gendered expectations’, ‘being bodily empowered’, and ‘altering gendered positions and stance to life’ point to empowering processes of change that allowed them to cope with distress, despite sometimes continuously stressful life situations. This intervention also decreased stress-related symptoms such as anxiousness, restlessness, muscle tension, aches and pains, fatigue, and impaired sleep.

**Conclusions:**

The participants’ experiences of the intervention as a safe and exploratory space for gendered collective understanding and embodied empowerment further indicates the need to develop gender-sensitive interventions to reduce individualisation of health problems and instead encourage spaces for collective support, action, and change.

## Background

Mental health problems are common during adolescence and emerging adulthood [1-4]. Worldwide, anxiety and depression are particularly frequent, with early onset in late adolescence and young adulthood [1,3,5]. The prevalence for mental disorders varies between the continents, but is at least 20% in the general population of 18- to 24-year-olds [3]. Sweden has recently been noted as having the largest increase in mental health disorders among young people in the OECD countries [6]. Almost 25% of 16- to 18-year-old Swedes suffer from mental disorders such as anxiety and depression, and unemployed or non-student youth aged 20–24 years have a 2.3 times higher risk than students and employed youth of being admitted to hospital due to depression [7]. In addition, over a third of all new claims for disability benefits in Sweden are attributable to mental health disorders among young people aged 16–24 years [6].

In recent years, mild or moderate mental health or psychosocial problems among young people have also been expressed as stress and stress-related problems [8-11]. ‘Stress’ is commonly understood as a condition where adverse and demanding circumstances exceed the individual’s ability to cope with them [8]. The term stress also covers external stress factors, so-called stressors [12]. Stress-related problems among children and young people are often interlinked with common health or psychosomatic complaints such as stomach- and headache, neck and shoulder pain, muscle tension, nervousness, and fatigue [11,13-16]. Wiklund et al. [11] also found significant correlations between perceived stress, for example, school pressures, and anxiety among northern Swedish adolescents 16–18 years old. In addition, stress and mental health problems during youth can be associated with psychosocial concerns such as family conflicts, sexuality, harassment or bullying, violence, suicidal thoughts, self-harm, and alcohol or drug use [2-4,10]. Such complex co-occurrences of mental, physical, and social problems influence young people’s needs for support and therefore place special demands on youth health services.

In addition, gendered stress and mental health patterns are observed in several countries, indicating that girls and young women 16–24 years old report more problems compared to boys and young men [5,6,17,18]. Similarly, several Swedish nationwide surveys have shown a tendency towards an increase in self-reported stress-related and mental health problems among girls and young women [8,10,19,20]. For example, in 2010 self-reported moderate or severe anxiousness, nervousness, and anxiety were estimated at 32% among girls/women aged 16–24 years compared to 9% in 1988 [21]. Moreover, in the age group of 20–24 years, the number of young women being hospitalised because of self-harm and depression has doubled between 1990 and 2010 [10]. Gender issues are therefore relevant to consider, both in research and in health promotion.

Consequently, it is crucial to develop both youth-friendly and gender-sensitive health interventions to prevent and capture mental health and stress-related problems at an early stage [4,6,22]. However, barriers such as stigma and problems in recognising symptoms have been identified as preventing young people from turning to health services, particularly for mental problems [4,22-24]. In Sweden, for instance, young people with these problems, and especially those who do not have access to school health services, seem to fall through the cracks of different support services – such as primary health care and child or adult psychiatry – because their problems do not entirely match the inclusion criteria of either service [25]. Thus, there is an urgent need, both in Sweden and elsewhere, to improve and develop feasible health services for this age group [3]. Muir et al. [26] point to the necessity of considering the heterogeneity of young people and making services available, regardless of age, gender, and culture. In addition, it is important to base support services on young people’s own ideas and formulated needs [27].

Worldwide, so-called youth-friendly health services are being developed to respond to young people’s complex health needs. According to the World Health Organisation (WHO) [28], youth-friendly services are characterised by accessibility, acceptability, and appropriateness. Social support, validation, and encouragement from others are aspects that are found to attract young people and facilitate their help-seeking behaviour [23,26]. In Sweden, youth clinics addressing sexual and reproductive health care are well-established, youth-friendly services. For many years, youth clinics have been offering services to young people, in the range from 13 to 25 years of age, nationwide. As a complement, there is a youth-friendly Web site [29] that, besides offering information and advice about relationships and sexuality, aims to provide a platform for young people’s discussions of oppressive norms and empowerment. Still, there are few Swedish youth health services specifically geared towards late adolescents and young adults with mental health problems.

Moreover, because young people’s mental health problems and help-seeking behaviour (in this article focused on young women suffering from stress) are found to be interlinked with social aspects of gender [30,31], it is important to take various aspects of gender and gendered living conditions into account when developing intervention models [32,33]. For instance, external stressors such as youth unemployment, educational pressures, normative body and health ideals, and sexual harassment seem to negatively influence young women’s mental well-being [10,31,33,34]. Studies also indicate that interpersonal stressors such as worries about family or peer relations are significant for young women’s experiences of distress and impaired mental health [9,31]. Other stressors affecting young women are changing living conditions in contemporary Western societies, including increased individualisation, and focus on consumption, fitness, and appearance [10,31,33,35]. This complex interaction between gendered external stressors and internal bodily stress reactions is an important consideration in health interventions geared towards young women experiencing distress [36].

Nevertheless, there are, to our knowledge, few Swedish youth health services and interventions that adapt a gender-sensitive approach to late adolescents and young adults with stress and mental health problems. Gender-sensitive interventions have primarily been geared towards adults with mental health problems, as well as within the field of young people’s reproductive and sexual health [37-42]. These studies show how gender-sensitive interventions can reach beyond traditional health programmes, because they take into account constraining gender relations, perceptions, and norms – which also may be barriers to recognising mental health symptoms and turning to health services in time. Strategies used in gender-sensitive interventions for young people have, for example, been to address gendered power imbalances and ‘double standards’ in heterosexual partner relationships [38,39], to be open to more complex gendered attitudes and perceptions among youth [38], and to empower young women to seek support in groups [40,41]. Empowerment is, in women-centred or feminist programmes for girls and young women, defined as a ‘process through which individuals are moved to act on their own behalf’, which aims to encourage their specific strengths and resources, including social and relational competences [41]. Even if there are policies and proposals for implementing gender-sensitive interventions and health plans worldwide, there are still barriers to implementing such strategies and services [43,44]. Gender sensitivity in health care presupposes a consciousness among health personnel about existing gender differences and how these are part of a sociopolitical and cultural context – a consciousness that includes decisions and actions in patient care [44]. In addition, the implementation of gender-sensitive health care has to be facilitated through a stronger understanding and connection among societal, organisational, and individual levels [45].

In Sweden, it is unusual for physiotherapists to work at youth health centres, as described in the present study, and in addition, physiotherapy is regarded as relatively ‘gender-blind’ [46]. To the best of our knowledge, no gender-sensitive interventions have yet been evaluated within physiotherapy. In Scandinavian and Swedish physiotherapy there are established and evaluated intervention methods suitable for adults with psychiatric disorders, as well as stress and pain management for adults [47,48], yet there are few studies on youth with stress-related and mental health problems. Such mental health studies with adults demonstrate that psychosomatically oriented and psychiatric methods, such as basic body awareness therapy (BBAT), have positive and long-term effects on pain, tension relief, and self-efficacy in individuals with non-specific musculoskeletal pain and psychiatric disorders [47,48], and inspire more positive experiences of body and self in women with fibromyalgia or chronic musculoskeletal pain [49]. Qualitative evaluations of group interventions with BBAT in psychiatric outpatient care and chronic pain rehabilitation for adults indicate that feelings of being included in a supportive group environment encouraged empowering processes that lead towards improved bodily functions and finding strategies for better health [49,50].

In this study, our aim is to illuminate young women’s experiences of participating in a body-based, gender-sensitive stress management group intervention by youth-friendly health services in northern Sweden. Our analysis also includes participants’ narrated and embodied processes of coping, action, and change.

## Methods

### Overall study design

This qualitative intervention study is a substudy of a larger research project, Stress and Health in Youth (Umeå SHY). The overall project aims to develop knowledge and understanding about stress and health among young people and to develop gender-sensitive intervention models. An additional aim of the research project is to integrate sociocultural and gender-theoretical perspectives as applied within medicine and health research. The overall project is being conducted in close collaboration with school health and youth health services, and uses a mixed-method design that combines qualitative and quantitative methods.

The gender-sensitive, physiotherapeutic group intervention model described in this study has been evaluated with both qualitative and quantitative methods. Before and after the intervention period, participants were interviewed [33] and answered a questionnaire consisting of questions about their stress, health, and bodily experiences. During the intervention period, participants kept short logbook notes. The current study is limited to analysis of the qualitative interviews conducted *after* the intervention period.

### Gender-theoretical perspective

Our gender-theoretical perspective, as applied in the overall project and in the intervention groups, is based on the understanding of gender as a social construction, where gender is produced and reproduced in ongoing social, cultural, and hierarchal relations and processes [45,51]. We are also informed by phenomenological perspectives on the ‘lived body’, in which gendered subjectivity is viewed as situated, experienced, and embodied through, and in relation to, historical, cultural, and social structures [36,52]. The gender-sensitive pedagogy used is more specifically described in the section Intervention model: stress-management courses.

Our previous analysis of the present sample suggests that stressors and demands in young women’s lives are multiple, and influenced by their social context as well as by social constructions of gender [33]. Such gendered stressors also are embodied and expressed in bodily symptoms and bodily dissatisfaction; they are linked to, for instance, problems such as negative eating and exercising behaviours [33,35,53]. Wiklund [36] highlights and problematises the close interlinks between discursive, embodied, and materialised processes of ‘doing stress’ and ‘doing gender’ that are expressed through a normative and constrained femininity. This furthermore points to the close interlinks between health and stress development and social aspects of gender.

In the present study, the gender-theoretical perspective was applied in the pedagogy of the gender-sensitive intervention and used as a critical and theoretical lens in analysing the young women’s experiences of participating in the course – mainly elaborated on in the discussion section.

### Research setting

The study was conducted at a youth health centre in Umeå, a university city in northern Sweden. The centre can be defined as offering youth-friendly services according to WHO’s definition [28], because it is easy to access and offers cost-free, multidisciplinary consultations. At the time of the study, the youth health centre specifically addressed young people, 16–25 years of age, with psychosocial problems and mental ill health. The centre opened in 2004 and was organised as a collaboration between the local county council, the municipality, and the employment and health insurance offices in the city. The initiative for the youth health centre originated from an identified need and political decision to capture young people with mental health and psychosocial problems in an earlier stage, and to offer a broad professional expertise gathered under one roof to cover young people’s different needs. Thus, the centre was based on multidisciplinary teamwork with a staff consisting of professionals such as a physician, nurses, psychologists, and social workers, many specialised in psychosocial problems and psychiatry among children and youth. In addition, a physiotherapist (the first author, MS) with specific competence in psychiatry, psychosomatics, trauma, and stress was part of the regular staff.

Since 2005, the youth health centre has also offered group counselling, in addition to individual support and team counselling. Between 2005 and 2009, stress management courses for young women were initiated, developed, and implemented at the centre as part of our larger research project, Umeå SHY. All the stress management courses (n = 7), consisting of eight sessions, each lasting about two hours, were led by the physiotherapist at the centre, who also worked as a research assistant in the research project and is the first author (MS) of the present article. The last author (MW), who initiated the research project, and the group leader (MS) were jointly responsible for planning the content and pedagogy of the stress management intervention. MW also functioned as a participant-observer during the first three group intervention periods. The second author (EBMO) has been a supervisor in the project since the start.

### Recruitment and participants

The stress management course was advertised at the youth health centre, the youth health clinic, the local employment office, and student health services at the university. Information about the course was also communicated to the target group through posters, leaflets, the centre’s Web site, and personal recommendations from the personnel at the youth health centre and the youth health clinic. Inclusion criteria for participating in the project were self-defined stress-related problems, girls and young women aged between 16 and 25 years, and interest in participating in a group-based course concept. Exclusion criteria were severe mental health disorders, such as psychosis, or other reasons that made group participation not relevant or unsuitable. The sampling procedure was convenient and consecutive, with participants contacting the centre directly without referral. All potential participants had a one-on-one meeting with the course leader, where participation was determined. Before the course started, participants were thus individually informed about the content of the course and about the research project.

In total, 55 participants joined one of the seven intervention groups that ran in 2005–2009. Of these, nine dropped out before completing the course. Dropouts primarily lacked time or had other difficulties scheduling the course. Some were offered other counselling or treatment for their problems. A few quit because the course concept did not meet their expectations. This article comprises 32 interviews drawn from the 46 participants who completed the whole course. Those interviewed represent all the seven intervention groups that were run during the project period. Of those 46 participants who fulfilled the course, 14 were not interviewed, the majority because of difficulties finding time with a tight work or study schedule. Some did not turn up for the interview at the agreed time, and a few did not want to be interviewed.

Those 32 participants who finally were interviewed were between 17 and 25 years of age (mean age 22). More than half of the participants were university, adult education or upper-grade students, while fewer than one in four worked full-time or part-time, and more than one in four were unemployed or on sick leave. The employed and not-employed participants were evenly distributed among the courses, even though most of them were students. Among the participants who worked, the employments were mostly temporary. Half of the participants had moved from other parts of Sweden to study or to work. Most of the participants were born in Sweden and none of them had children. See Table 1 for a description of the participants.

**Table 1:** Characteristics of participants in the stress management intervention

**Age in years (number)**	**17–19 (3)**	**20–22 (19)**	**23–25 (10)**
**Living situation**			
With parents	3	5	
With partner/friends		4	3
Alone		10	7
**Level of education**			
1^st^–9^th^ grade	3		
10^th^–12^th^ grade		11	
University*		8	10
**Present occupation**			
10^th^–12^th^ grade school	3		
University studies		6	7
Adult education		3	
Work – fulltime		3	1
Work – part-time		2	
Unemployed		3	1
Sick-leave		2	1

*10^th^–12^th^ grade and a course, or programme, at the University.

### Intervention model: stress management courses

The intervention model was announced as a stress management course led by the physiotherapist at the youth health centre and linked to our research project, Umeå SHY. Each intervention group was limited to a maximum of ten participants, the average being six, and all were similar in content and form, although the participatory course concept allowed some flexibility within pre-defined frames. The concept was a combination of well-established and evidence-based methods used in Scandinavian physiotherapy, primarily basic body awareness therapy and progressive muscular relaxation [54]. The pedagogy was informed by group counselling [55], and a problem-based physiotherapeutic pedagogy, combining reflective discussions and BBAT, originally developed for adult women with psychosomatic and long-term pain problems in primary health care settings [56]. In addition, a gender-sensitive approach was utilised [41,45].

BBAT is a resource-oriented and holistic ‘body-mind’ physiotherapeutic method aimed at strengthening and restoring essential functions of the physical self [57,58]. Central tenets in this body-based concept are to facilitate grounding, stability, balance, flow of movement, breathing, and mental/cognitive awareness and embodied presence. In BBAT, emotional, physiological, psychological, and existential dimensions of bodily experiences are viewed as essential and vital parts of the total identity. Lundvik-Gyllensten et al. [57] express this in terms of ‘embodied identity’. Being aware of and attentive to movement and bodily presence is central to rediscovering bodily resources and abilities, guidance that to some extent is similar to mindfulness techniques used to reduce stress [59]. The role of the leader is to guide participants to explore, experience, and integrate sensory and affective dimensions of the body, and also to facilitate reflection and verbal expression of bodily experiences [58]. Overall, this body awareness philosophy formed an important base in the stress management course.

The participatory, problem-based, and gender-sensitive approach integrated into the course concept was based on our knowledge of young women’s gendered living conditions and our gender-theoretical understanding of gender as being created in social contexts and relations [33], as described in relation to the overall research project above. In line with feministic frameworks the gender-sensitive approach aimed to empower girls as a group to listen to and base the content on their own thoughts and experiences, and to facilitate a salutogenic approach to their bodies [41,45,60]. The group leader thus functioned as a facilitator and directed participants to adopt less individualised views of their perceived stress. The group-pedagogy and the gender-sensitive intentions implied being sensitive to the young women’s expressed needs, both in the choice of themes for discussion and during the body-oriented parts of the sessions. Themes elaborated on during the course were identified during the initial group sessions, or originated from the research team’s knowledge about young women, gender, and stress, but had also emerged from the young women’s own narratives told to the group leader and researcher in the one-on-one meetings or interviews before the course started. In addition, the group leader – during ‘mini-lectures’ – contributed to general or specific knowledge and facts about stress and stress physiology, including the importance of rest and restoration [12].

More specifically, the design of each session included reflective discussions, short general lectures on stress and stress reactions, BBAT, and relaxation. Discussions were based on questions like ‘How do I react to stress?’ ‘What is stress for me?’ ‘How do I cope with stress?’ ‘What is it like to be a contemporary young woman?’ and ‘How can I set limits?’ Themes were also thought up in each group and might address pressures in life such as sociocultural and gendered norms, body ideals, and dieting, pressures of perfection and high achievement, and expectations for girls to assume responsibility in social relationships and in caring for others’ needs.

### Qualitative interviews

All of the interviews took place at the youth health centre, soon after the end of the course. The interviews were thematic and based on open-ended questions such as ‘Please, tell me about your experiences of the course’, ‘How do you experience stress and life now?’ ‘What was most important in the course?’ ‘Have you experienced any changes?’ and, in that case, ‘In what way?’ Several of the interviews also touched upon bodily experiences, emotions, and relationships. Both MS and MW conducted the 32 interviews. The interviews were 20 to 70 minutes long, and for the most part lasted about 45 minutes. All interviews were recorded digitally and transcribed verbatim.

### Analysis

The interviews were analysed using qualitative content analysis according to Graneheim and Lundman [61]. The analysing procedure was inductive and followed a stepwise process in which all authors were involved, with the first author (MS) mainly responsible. Qualitative content analyses include both manifest and latent content. First, each interview was read several times to obtain a sense of the whole and to identify the meaning of the participant’s experiences. Next, MAXQDA software [62] was used to aid in sorting and grouping the manifest content of the text into elements and codes on a descriptive level. In this grouping procedure, meaning units were identified and labelled with a code, which then were sorted into two content areas: ‘experiences of the course and changing processes’ and ‘ongoing problems and negotiations’. A content area is a rough structure of content that is helpful when looking over the material as a whole and can be identified with little interpretation [61]. The condensed meaning units and codes were then interpreted and compared for differences and similarities on a slightly more abstract level of understanding. This analysing stage involves a forth-and-back movement between the whole and the parts of the empirical data [61]. Thereafter, the latent contents of the analysis were formulated into five tentative themes. Themes were discussed, processed, and reflected on before the authors finally agreed on a set of seven subthemes, three themes, and one main theme (Tables 2 and 3). Qualitative content analysis, as applied in this study, holds to a relatively descriptive level – close to participants’ own words and expressions.

**Table 2:** The process of the analysis, moving from the text (meaning unit) to codes, subthemes, and themes

**Meaning units**	**Codes**	**Subthemes**	**Themes**
I have learnt of many good ideas and so on, and also talked with others	Recognising oneself in other girls		Finding a social oasis to challenge gendered expectations
		Being confirmed in a non-judging and supportive atmosphere	
	Not being alone		
Before, I only focused on just one thing, but now I have been able to see that, yes, but perhaps it is this and this too, and then it’s somehow easier to deal with it	It is not my fault		
	Seeing causes and connections	Making space for reflections on gender and stress	
It’s like a fear of failure and not being good enough, which is very typical for many girls	Feeling pressures and inequalities (as girls)		
	Reduced symptoms		Being bodily empowered
I listen more to how my body feels. My body reacts directly if it becomes difficult	Listening and understanding bodily signals	Approaching the problematic body	
I have a better relationship to my body now	Feeling trust and being conscious of the body	Finding breathing space	
It is, as you are stopping, a very concrete stop and like really relax on the floor and try to drop all these thoughts	Time for silence and reflection	Upgrading oneself and one’s abilities	
	Valuing oneself		
Like, you have come to realise that everyone else is not better	Thinking more positively		
I have stopped seeing problems that do not exist	Slowing down the pace and prioritising needs	Switching pace in life	
I have more energy to do more things, but at the same time I have not quite that many things to do any longer	Releasing energy and decreasing demands		Altering gendered positions and stance to life
It’s really important to stay away from all that and try not to compete. You cannot be coolest, not be prettiest, or never be the best at everything	Managing and resisting (social/gendered) expectations	Setting limits and resisting outer pressure	

### Ethical approval

Written and verbal information about the study was given to the participants in several steps before the course started, first by telephone and then in conjunction with the first interview. The participants were informed that participation in the research project was voluntary and that they could withdraw their participation at any time without giving a reason. Participants were assured of confidentiality in the project and anonymity in the presentation of final results. After the stepwise information, participants gave their written informed consent. The personnel at the youth health centre were well informed about the research project, and were prepared to offer individual counselling if participants demonstrated significant distress. Ethical approval was confirmed by the Ethics Committee of the Medical Faculty of Umeå University (Reg nr 05-045M).

## Results

Our analysis resulted in one main theme of ‘a space for gendered and embodied empowerment in a hectic life’, which together with related themes and subthemes illuminates how the young women in the study experienced participation in the stress management course as encouraging processes of change in the direction of understanding and managing stress in new or alternative ways (Table 3). The contents of the group intervention, requiring active participation, support, reflection, and bodily presence, strengthened their own resources to meet and handle problems of stress.

**Table 3:** Main theme, themes, and subthemes in the result

**A space for gendered and embodied empowerment in a hectic life**		
**Finding a social oasis to challenge gendered expectations**	**Being bodily empowered**	**Altering gendered positions and stance to life**
Being confirmed in a non-judging and supportive atmosphere	Approaching the problematic body	Upgrading oneself and one’s abilities
Making space for reflections on gender and stress	Finding breathing space	Switching pace in life
		Setting limits and resisting outer pressure

The main theme was thus generated to describe participants’ processes of empowerment, in which the content of the each theme builds on and is interlinked with the others. The theme ‘finding a social oasis to challenge gendered expectations’, together with the theme ‘being bodily empowered’, forms a prerequisite for the theme ‘altering gendered positions and stance to life’. Altogether, the themes create ‘a space for gendered and embodied empowerment in a hectic life’. Gendered and embodied empowerment emerged for participants through forming supportive relationships with other young women in similar stressful and strenuous life situations, through reflecting and giving voice to central experiences connected to stress and strain, and by starting to scrutinise and question gendered and normative expectations. Importantly, empowerment also embraced emotional and bodily experiences. This empowering process strengthened participants’ own resources to handle their perceived stress, although they still, after completing the course, had to face ongoing stressors and challenges – sometimes out of their own control. In the following, we describe the themes separately in more detail, supported by quotations illustrating how our analysis is grounded in the interview material. As the experiences and processes are complex and interlinked, there may be overlaps between the themes or interview excerpts.

### Finding a social oasis to challenge gendered expectations

The theme ‘finding a social oasis to challenge gendered expectations’ represents the participants’ narrated experiences of social togetherness and community through a process of sharing life stories in discussions with other young women in similar situations. Sharing experiences about stress as a complex and multi-faceted phenomenon gave them valuable insights into how various stressors and individual stress reactions could be related to social creations of gender and external demands from society in general, as well as in relation to their own everyday lives. The stories also revealed a process by which socialising with others, confirmation, and recognition were seen as reducing the burden of guilt and personal responsibility for one’s own situation.

#### Being confirmed in a non-judging and supportive atmosphere

To be critically evaluated and judged or even belittled, either by others or by oneself, was described as part of young women’s everyday life. In line with these experiences, many of them felt it troublesome to seek help for stress-related problems, as they had associated this with personal weakness and failure. Together in the group they experienced considerable relief and confirmation upon realising that they were not alone with these problems. This sense of relief that emerged from sharing experiences and identifying with one another also contributed to feelings of hope for change. Feelings of volunteerism and community within the group were experienced as reinforcing and revitalising. At the same time, taking the initiative to apply for the course was perceived as a mark of strength:

The only thing I thought was difficult with the stress course was to mentally admit to myself that I needed it. Sometimes I could feel like this, ‘Oh my God, what am I doing here?’ At the same time I felt an enormous appreciation of being there, so it was really difficult to digest, actually. In the end I found that I could relate to the others in the group, just because they had also slipped through the back door. It’s not about that we are worse or weaker people, but rather we have taken a responsibility for something that’s a big problem, and done something about it. So cred to us. (Student, 22 years)

This undemanding and permissive atmosphere in the course helped them to take part in confidential discussions about their experiences. A central aspect was the possibility to be seen and heard without being judged or feeling the need to deliver and perform. This supportive environment made it possible for them ‘to be themselves’, instead of having to pretend or keep up an appearance of perfection. Within this non-judgmental atmosphere they got valuable insights into how external stress affects one’s individual situation. Diffuse or taboo thoughts and feelings were unravelled.

I think we have had really good discussions within the group and talked about things that individually we might not have dared to talk about, perception of our bodies or whatever. . . Yes, and then in some way to be able to say, ‘I don’t feel good about this.’ And that’s been really good, because you wouldn’t dare to say it to someone else, haven’t even dared to acknowledge it to oneself. But when other people say it, when they dare to say it, then I can really recognise myself. So I think this has given me really a lot, I’ve learnt a bit more about myself and, yes, learnt to recognise my kind of stress and what triggers it. And that’s what it’s about, meeting in a group: where everyone has the same problem, then everybody dares to raise issues. That’s really great. Nobody judges anybody, no way. (Working, 20 years)

In addition, and as seen in the quote above, participants ‘learnt about themselves’, gaining new insights and concrete knowledge about stress and its potential causes, as well as learning about their own ‘triggers’ and stress responses. This also exemplifies how ‘meeting in a group’, enabled identification and giving voice to unspoken themes related to these young women’s experiences of stress, and how the collective process of recognition and confirmation contributed to feelings of strength and empowerment on both the individual and the relational level.

#### Making space for reflections on gender and stress

During the course gender-related stressors and norms were brought into view and challenged by such discussions as ‘being a contemporary young woman’ or ‘sexualised bodily ideals’. The group sessions gave ample space for self-reflection and deeper insights about gender and stress in a more general sense. The participants brought to the surface their ideas about ‘girl stress’, and to some extent, were able to challenge these from social and relational points of view. By comparing similarities and differences in one another’s stories, they experienced the ability to question and find nuances in perceptions such as ‘everyone else is perfect’ and ‘everyone else feels good’. Gender-related norms and ideals were thus scrutinised and problematised. In this way feelings of anxiety about being ‘different’ or ‘mentally ill’ could be decreased. On the other hand, the young woman below exemplifies how new insights helped her to understand that the stress and self-accusations she experienced were masking prolonged pressure.

For me it was sufficient to understand that it wasn’t only just stress (which I thought previously) but also that it was about a prolonged stress that had been ongoing for, well, maybe many, many years since I was small, or just a child. And what I previously thought was that I couldn’t achieve anything. I was accusing myself a lot, really, and that sort of thing about not getting things done, but now I realise that this was because I was sick, or depressed, or however I should put it. (Student on sick leave, 22 years)

The general opinion in the group was that ‘young women’s stress was more complicated in comparison with young men’s stress’. For example, they put forward how young women have to relate to complex norms concerning perfect appearance, high achievement, and social status. They discussed how unequal conditions resulted in young women experiencing greater pressure and being ‘expected to perform better than young men’ in order to be granted respect by society. Gender-associated relations and competition within groups of young women were also problematised.

Fora similar to the course were requested by the participants, in which girls and young women should be able to meet and support one another, in contrast to contexts reinforcing the more usual perceptions that women ‘don’t trust’ or ‘compete’ with one another. One participant expressed this, as ‘girls need to strengthen each other as a group’. These reflections and discussions led to perceptions that young women’s stress was not only a problem for them as individuals, but also was dependent upon gendered social relations, norms, and structures in the society in which they were immersed. Hence, this also led to widened or alternative understandings and explanatory models of perceived high pressures and demands – which served to unburden the sense of individual fault and failure.

### Being bodily empowered

At the start of the course, the participants told about their suffering from the physical, emotional, social, and existential consequences of stress [33]. The theme of ‘being bodily empowered’ therefore represents experiences from which they began to approach their problematic bodies. By engaging in body-based awareness and reflections, they achieved consciousness of how bodily sensations and reactions were strongly connected to the stress of their everyday lives. The course created space for relaxation and corporeal presence, which contributed to feelings of calmness, strength, and relief.

#### Approaching the problematic body

Before the course began, the young women experienced a conflicting and ambivalent relation to their bodies. These were seen and experienced as ‘problematic’, expressed by restlessness, sleeping difficulties, aches and pains, and resignation, and also as a general dissatisfaction with their bodies. Few experienced the body as a positive resource. Instead they were engrossed in bodily deficiencies and difficulties in fully controlling and mastering their bodies through training loads and food intake. In contrast to this, the body awareness sessions created a bodily space where participants instead could approach and explore their bodies as potential resources. For instance, the young women explored how being mentally present and paying attention to the body might influence unpleasant bodily sensations and overwhelming stress reactions. Some of them experienced the way feelings of anxiety, tension, and unpleasantness could be altered to become experiences of relief, comfort, and pleasantness. For most of them, relief and increased awareness of physical flexibility positively influenced concentration, sleep, and pain. In addition, the young women’s discovery of their flexibility and re-configurability strengthened their feelings of hope for change.

I’ve actually been able to sleep, that’s to say I’ve been able to drop off quite quickly recently and I’ve not had so many headaches. (Student, 23 years)

I haven’t had my usual difficulty going to sleep and that stress about the future, which I experienced previously. Well, now I’ve been able to relax a bit within it and try to feel that things are going to work out, anyway. (Working, 20 years)

During the course, participants also explored balance and stability in different positions, which further strengthened their sense of confidence and flexibility. As an example, one young woman described how her feeling of grounding and balance in her body helped her to meet a challenging task, instead of experiencing herself as ‘wilted and crushed’. This heightened stature improved her self-esteem and her ability to react in ways different from her usual reactions.

Approaching and experiencing closeness to the body also involved inter-relational physical contact and touch through massage in pairs, during which participants experienced giving and receiving touching. This was experienced as calming and confirming. The massage was appreciated, even though initially some participants were cautious about such close contact with others in the group. This was related, for example, to personal integrity, self-disgust, or earlier experiences of bodily insult. Nevertheless, close interaction and physical contact with oneself and other young women helped them to a greater acceptance of themselves and their own bodies:

I’ve learnt a great deal. My body has, in a way, its own feelings. Sometimes I can feel in my body how I actually feel in my head, or how I feel mentally. I’ve also learnt that I can relax and feel that ‘now I’m relaxing’ . . . heightening my awareness. (Student, 22 years)

As expressed by the young woman above, understanding of bodily responses and signals led to a positive consciousness of the body and its close relation to emotions, desires, and well-being – or rather to a sense of the ‘emotional body’ as an integrated part of the entire being. This also points to affective and sensory dimensions of the participants’ experiences of their bodily being, as well as of their well-being. In short, BBAT and relaxation training served to reduce stress and ‘wind down’ their restless bodies, and thus to moderate the physiological and affective arousal that were part of their stress responses. In addition, it helped them to embrace their bodies and physical experiences as positive resources, and as part of wellness and being at ease – in contrast to experiences of living under constant fear or pressure.

#### Finding breathing space

On the whole, the young women experienced the course as a ‘breathing space’ in their hectic and demanding lives. On a more concrete level, paying attention to their own breathing involved awareness of bodily rhythm and tempo. They discovered that calmer and more open breathing reduced their stress and created space for well-being.

There’s been a certain amount of change. Previously I had more, yes, a lot more heart palpitations, and I had very, very tense, shallow breathing. Now it’s gone down a bit, at least to my stomach, and I’m beginning to breathe a bit better. I’m not as tense and stiff as I was previously, more relaxed now, as it were. Anyway, that’s what I believe. (Student on sick leave, 22 years)

Another participant described how, by concentrating on her breathing, she was able to cope with pain and stressful situations with calm, instead of reacting with panic and feelings of impending catastrophe. In addition, the breathing space that was created facilitated experiences of corporeal presence*,* expressed as ‘being here and now’, ‘to feel’ and to have access to the body. The exercises were done in silence and gave the participants an opportunity for reflection, which felt liberating for many of them. Their bodily awareness and inner peace eased the requirement of always having to be ready to achieve and perform. To get ‘undemanding’ time for oneself and rest from having to relate to others became an important oasis for self-reflection and recuperation. It also worked as a way to rest from daily worries, external demands, and their own critical self-evaluations.

At first I had lots of problems when it was, like, ‘Next time we will just do exercises’. Well, then I felt ‘Oh my God’ and panic, ‘How am I going to fix that?’ Then as I became more and more secure with the exercises I felt it was really nice not to talk so much. Instead, the exercises gave one a bit of time for oneself. So every Wednesday became a two-hour oasis when one, I mean, I could slow down and learn how things were feeling inside my body. (Student, 22 years)

Taken together, their stories express a process of moving towards body-anchored confidence in oneself and bodily freedom – which is our interpretation of bodily empowerment*.* These narratives expressed dimensions of emotionality, presence, grounding and stability, and interpersonal interaction, which all seemed to be prerequisites for action and change.

### Altering gendered positions and stance to life

The theme ‘altering gendered positions and stance to life’ represents experiences of progression in the young women’s ability to handle and manage difficult stress situations. Hence, the course was used as a forum to challenge oneself, to try out new positions and skills, and to develop strategies and competence. Processes of positive reinforcement resulted in improved feelings of security and confidence. This can be viewed as the beginning of altered gendered and social positions.

#### Upgrading oneself and one’s abilities

At the start of the course, the young women’s self-perceptions were characterised by feelings of anxiety, uselessness, and diminished dignity. In time, a sense of bonding with the other participants and a perception of lowered demands on performance and expectations resulted in a gradual re-evaluation of judgements concerning themselves and their own worth. By a process of confirming and praising one another and their abilities, they were able to create space for thoughts such as ‘You’re good enough as you are.’ This more relaxed and changed stance towards oneself also included less focus on perfect body, weight, and appearance – although such pressures were still problematic to handle. In this way they could create nuances in perceived difficulties and failures in everyday life. Statements such as ‘It’ll work out’ or ‘It’s not the whole world’ became more and more common, and the future was seen in a more positive light.

Previously, to a tremendous extent my thoughts would go on and on about how I diminished myself all the time, how weak my self-esteem was, and that sort of thing. Just as in every situation I felt bad or other people thought that I was bad. Well, now things have changed a great deal. Naturally, it’s not disappeared completely, but this used to happen every day before, and nowadays maybe I think that way once a month or so. (Working, 25 years)

This positive upgrading and re-evaluation of themselves and their abilities resulted in energy and endurance, feelings of well-being and calm. Importantly, their re-evaluation included less individualised views on their stressful situations.

#### Switching pace in life

The relation to time and time pressure was central to the young women’s perceptions of stress, and thus ‘switching pace in life’ was a central theme in the process towards altered strategies to handle pressures and demands. The lives of the majority of the young women had a rapid tempo, with high levels of ambition in many parallel areas, and experiences of limited time. The combination of reflective conversations on stress in daily life and the self-reinforcing bodily attention during the course helped them to question the tempo of their lives, as well as the unreasonable demands from both themselves and others. The course created space in which they could slow down and adapt their tempos to their own rhythms, instead of what some termed as ‘running away’ from oneself.

I can really listen to, I’ve time to listen to ‘what do I really want?’ and not just go along with things – you know, it’s a real stress to feel that I’m going along with things that I don’t really want. That’s something I’ve often done. It wasn’t me that decided – I just followed along and then I got really stressed out about ‘was this what I wanted?’ and that sort of thing. And now it really feels that I’m stronger. (Working, 20 years)

Thus, as expressed in the quotes, they highlighted the importance of being able to give priority to one’s own needs and choices instead of allowing oneself to be ‘sucked into’ the ‘screwed-up’ tempo.

Yes, well it feels like I have cut down on the pace of life. Earlier, I felt that I had to fill my day with many activities . . . I have, like, cut it down and I think a lot about what I have to do today, what is the absolute most needed to do today. So I, like, prioritise between what I need and what I want. (Student, 22 years)

Switching and slowing down the pace was also described as a sense of getting ‘more time for oneself’ and having time to ‘take care of’ oneself, in addition, representing a shift in focus and priority. Overall, trying out a new – often lower – pace and loosening up tight schedules resulted in an increased sense of freedom to move and act differently, which unburdened them from pressures and positively affected their well-being.

#### Setting limits and resisting outer pressure

The young women continually recounted that external and gendered pressures, for example, high demands regarding perfect appearance, a slim and fit body, performance at school, and caring for others, were heavy and difficult to resist. Through experiences of heightened self-awareness and alternative social positions during the course period, they were able to establish boundaries in relation to their families and to others in their proximity. This could involve ‘leaning back’ in order to avoid being devoured by taking responsibility for other people’s problems – which was not always very easy. They found it difficult, however, to stand up and gain respect for their decision to set boundaries with respect to their social contacts.

I’ve tried to resist accepting too much responsibility. But it’s a bit half-hearted. . . When I try to resist giving a helping hand, in the end I find myself anyway interested as to how things worked out . . . so I get landed with responsibility again. . . Not everything is my concern, although actually it’s clear that things do get under my skin if it’s friends that have problems or if something has to be arranged, a party or a movie night or whatever. Then I get involved, but I don’t have to do everything. (Unemployed, 20 years)

As described above, prioritising one’s own need for solitude and relaxation before the need to care for others or socialise was especially challenging, because the latter were perceived as having a higher status. Another social position of ‘claiming space’ and raising one’s voice in social contexts could also meet with opposition – because the social norms of femininity demanded retreat and diminishment in order to gain acceptance. Nonetheless, the young women experienced that their struggle to set boundaries and resist external demands had importance not only for their own situation; it could also generate respect in their social relationships.

We’re trying to find new ways of understanding each other, but I can see that I’m being exposed to enormous pressures when I choose new paths, and these come from family as well. For example, setting boundaries. I’ve got to persist, and that may be perceived as ‘how difficult everything is now’. But I do see that things are really positive, and more so when I get positive feedback. At first, I feel anger, but then comes the positive bit, that they respect me. (Student, 22 years)

The described processes of negotiated and altered gendered positions reflect bodily and personal empowerment embracing the possibility of ‘taking a step forward’, ‘claiming space’, ‘leaning back’, or withdrawing from negative or demanding attention in both a physical and metaphoric sense.

To summarise, the young women experienced the stress management course as empowering and reviving, although it was inevitable that they still had to cope with continuous stressors in life. The young women’s choice to participate in the stress management course offered them an opportunity to pause and to reflect on their stressful and demanding life situations. Our analysis illuminates how sharing experiences in a supportive and trusting atmosphere encouraged them to mobilise and expand their space of action by being encouraged to question and renegotiate gendered performances related to normative and constraining feminine ideals. Embodied experiences of relief and flexibility made it possible, at least to some extent, to be released from individualistic expectations and pressures of success and perfection, as well as from gendered burdens linked to social and relational responsibility, self-criticism, and guilt. In short, the intervention model can be said to represent a safe and explorative, individual and collective space, which facilitated gendered identity work, social support, and confirmation. It also represents a bodily space, as well as a breathing space, that served to decrease stress symptoms and supported the discovery of bodily resources such as strength, stability, and relaxation.

## Discussion

In this qualitative study, young women reflected on their experiences participating in a gender-sensitive, health-promoting group intervention, known as a stress management course, at a youth health centre in northern Sweden. The overall results of our analysis suggest that the evaluated stress management course facilitated ‘a space for gendered and embodied empowerment in a hectic life’, implying that it both contributed to a sense of individual growth and unburdened the participants of stress problems within a trustful and supportive context. The experiences narrated by participants after completing the intervention period also point to processes of change and altered strategies in coping with distress, despite sometimes continuously stressful life situations and ongoing problems. Based on the results, we conclude that group intervention helped to empower these girls to cope, act, and change within the given social and gendered constraints. In the following we discuss the experiences explored and the processes of change mediated by the participants, formulated as *needs* and *uses* that the intervention addressed.

### The need/use of social support and gendered collective understanding

The theme ‘finding a social oasis to challenge gendered expectations’ points to the importance of being in a supportive social context, particularly when one’s life situation is strenuous and stressful. The trusting atmosphere was facilitated by the group pedagogy and the contacts with others in a similar situation. Hence, this intervention – when understood as a social oasis – contributed to the young women’s well-being and empowerment in different ways. In this context, we refer to empowerment mainly on individual and relational levels [41,63]. This is also in line with Kelly [41], who highlights the empowering aspects of offering young women the possibility to form relationships with other women, express their own voices, and become comfortable with their bodies in groups with other women. Perhaps the most beneficial aspect is the strong feeling of social support from ‘similar others’, which in our study refers to other young women experiencing similar pressures and demands. This is in line with Thoits [64], who emphasises the value of social support from similar others. Such specific support is defined as an effective *buffer* against stress and mental ill health in terms of empathic understanding, validation of feelings, coping encouragement, and inspiring hope. A sense of coherence also may function as a potential moderator between stress and subjective health complaints [17]. In addition, recent behaviourally oriented stress research emphasises the role of social activities and relationships as a buffer to stress [65].

Thus, being confirmed by and identifying with similar others seemed to be key to participants’ gradual ‘upgrading’ of themselves and their skills and abilities – as well as to the central theme of ‘altering gendered positions and stance to life’. Similar positive outcomes of the mutual support, enhancement of self-efficacy, and empowerment experienced are found in other studies of group-delivered self-management courses and self-help groups with individuals suffering from mental illness or various long-term health conditions [66,67]. Empowerment and enhancement of self-efficacy is seen furthermore as central to behavioural group interventions designed to promote ‘self-management’ of health and life [66]. On a more general level, social support is associated with positive health [64,67,68], and there also seem to be links between empowerment and health [69].

The subtheme ‘making space for reflections on gender and stress’ highlights the importance of a shared understanding of young women’s stress as a complex phenomenon comprising multiple socially shaped pressures and demands as well as gendered self-identities. We believe that these *shared insights* helped participants to re-evaluate and challenge these expectations and to discover alternative ways of understanding and coping with their own situations. The observed results of *new and widened explanatory models* point to the development of *collective understandings* and a *shared identity* that the young women gained from the supportive group setting [41,66]. The concept of ‘collective wisdom’ [70] captures the advantages of problem solving and reflecting together with similar others in a group. Participants’ new and widened explanatory models and shared insights seemed to reduce their sense of personal responsibility for their stressful life situations, including feelings of individual failure connected to distress and seeking help. Similar processes of collective understanding and experienced support are reported from interventions guided by a feminist framework in which women can empower one another to cope and eventually change adverse life situations [40,41]. All responsibility for change cannot be placed on girls’ and young women’s shoulders, however.

### The need/use of safe and explorative spaces

The course as a social oasis, in addition, can be interpreted as a safe and exploratory place for identity work and negotiations of femininity. It was interesting to see how participants encouraged one another to reflect on, and challenge, gender norms. Youth researchers such as Sveningsson Elm [71] and Oinas and Collander [72] argue that girls need and benefit from these safe places or ‘girl rooms’, where they can try out and challenge their social and gendered positions. These safe places and girl rooms – in the present study, like similar others – may counterbalance the negative external pressures that girls and young women appear to experience. They may also strengthen and protect their self-respect and personal integrity. Our earlier analysis of perceived stressors within the same sample of young women points to the perceived demands to conform to an individualised normative femininity of perfection and high achievement with respect to the care for others [33,73]. Similarly, Oinas [74] problematises the sexualised social positions that girls and young women negotiate. Based on participants’ positive experiences of coming together in a supportive group, we hypothesise that interventions like this – when understood as a safe and explorative space – also may serve an important function in terms of reducing stress.

In addition, the body awareness practice offered a safe, reviving, and *explorative bodily space*. The theme ‘being bodily empowered’ emphasises the value for these young women with stress-related problems of getting professional guidance in how to understand and cope with the strenuous relationships with their bodies, including severe stress symptoms. We believe that participants were able to approach a problematic, tensed, and painful body in a stepwise process. Within this space, the young women’s corporeality gradually became associated with well-being, instead of suffering. The body came to represent a positive resource, rather than merely a problem and burden. This way of gently exploring the symptoms and not being afraid of uncomfortable sensations has been found to be a powerful treatment principle in stress and trauma therapy [75]. In addition, the utilised elements of awareness and nonjudgemental acceptance of one’s experiences have been shown to have positive effects on psychological distress and a range of psychosomatic conditions [59]. BBAT, as applied in the intervention, also served to decrease physiological and affective arousal, and thus positively influenced and moderated the sympathetic nervous system and related stress symptoms. Sensory and affective dimensions are also seen as central in the phenomenological understanding of bodily connectedness and embodied subjectivity, which is part of human intentionality to interact with the environment and the world [52,76,77]. We also understand this embodied process of approaching and experiencing ‘the emotional body’ as a process towards what the phenomenologist physiotherapist Rosberg [77] defined as a sense of being ‘at home’ in oneself and in one’s body and life (Swedish: *hemmastaddhet*), which in turn represents bodily and existential trust, a sense that in this sample was interpreted as impaired [36]. Experience of bodily connectedness, wholeness, and being at home in one’s body and life is viewed as an important dimension of health and well-being [50,77,78].

Another interesting result of participants’ processes of bodily empowerment is their experiences of stability and embodied trust. Our results indicate that the participants within the explorative bodily space that was created during the group sessions were offered an opportunity to explore stable and flexible body positions. Particularly, their experiences of bodily stature and claiming of space seemed connected to their self-reliance and self-image. Through and from these new, gendered body positions the young women could not only experience personal strength and integrity but also challenge and try out strategies of ‘setting limits and resisting outer pressure’. We believe that the combination of reflective discussions and experiencing the body in a holistic and salutogenic manner contributed to increased awareness of the importance and protection of a *space for one’s own needs*, at the same time as it facilitated and extended *space for motion and action*. Similarly, Gustafsson et al. [49] described how women suffering from pain improved their self-image and the ability to set limits in social relations in a process of shifting from ‘shame to respect’, as a result of their participation in a body awareness treatment intervention.

Other positive steps in participants’ processes of moving towards embodied empowerment are their experiences of ‘finding a breathing space’. On the more abstract level the intervention – when understood as a breathing space – can serve as a metaphor representing their experiences of the course as both a safe place, as described above, and a space for reflection. *The need and use of breathing space* is then also connected to a change of pace in life. If we, in contrast, turn to the more material and intra-individual level, it is well-known that breathing is essential for human life and vitality, and can therefore also play an important role for recovery and well-being [79]. Furthermore, breathing rhythm is directly interwoven with tensions and emotions in the body, and is thus responsive to stressors and strain in life [57].

From our analysis, it is clear that participants experienced excessive pressure on their time and a problematic relation to time and the forced tempo of life [33]. However, the thematic of the group sessions encouraged them to influence this negative experience by ‘stopping time’ and slowing down the pace. The participants started to reflect upon and prioritise time in a slightly new way. Thus, ‘switching the pace of life’ became a strategy to resist external pressures.

### The need/use of (collective) change and action

The theme ‘altering gendered positions and stance to life’ highlights how participants used their released vitality and self-reliance to explore new ways to handle stressors in life. We believe that this group pedagogy created a space for different individuals and ‘multiple femininities’ to feel comfortable and safe enough to explore altered embodied gendered positions to manage stress and resist or challenge outer pressures. Nevertheless, our follow-up interviews revealed that participants differed regarding the extent to which they were able to transfer insights and experiences from the intervention into practice and action in their daily lives. The participants’ ability to transfer gained insights and incorporate changes into daily life after participating in an intervention is a crucial point for more stable behavioural changes [80]. Our analysis indicates that participants took important steps towards changes that were possible within their present social life situation and given gendered constraints. However, we believe that several of the participants would have gained from some kind of follow-up, extended social support, or ‘booster’, to continue already initiated processes towards more sustainable change and improved well-being.

Thus, our analysis highlights how the young women we studied continually struggled with ongoing problems and negotiations related to gendered stresses in life. They worked hard to protect themselves and to resist internal and external pressures and gendered expectations. In line with findings from other studies, the young women in the present study struggled to relate to conflicting ideals and feminine subject positions [33,53,74]. For example, Gustafsson et al. [53] found that young women with eating disorders handling social pressures often chose to follow others’ expectations instead of following their own needs, even if the latter strategy was the most desirable. Similarly, Liimakka [81] observed that even ‘feminist’ women, despite their consciousness of gendered ideals and expectations, had difficulties protecting themselves from feeling dissatisfied with their bodies and appearance. Therefore, Liimakka [76,81] suggests that young women need to develop ‘bodily empowerment’ and ‘embodied agency’, which we understand as a power to act, grounded in embodied trust and acceptance of self – and this is in line with our conclusions in the present study. However, this still represents a quite individualised view of young women as responsible for handling problems and stressors attached to a wider societal context. It is important to acknowledge that young women themselves cannot be expected to change all the outer conditions that have to be acted upon politically and collectively.

### The need/use of gender-sensitive youth friendliness

The stress management intervention described was a single intervention conducted within the context of youth-friendly health services [28], which we believe serves as an important frame when evaluating this specific intervention. The youth centre, with its safe and sensitive milieu, seemed to help induce the young women to apply for the course, because the majority of them applied there as a first stage to seek help for their problems. Earlier studies have indicated the need of such youth-friendly services to capture and encourage young people to seek timely help, and also to complete the interventions started [23,26,27]. In addition, participatory research, such as applied in this study, is recommended in the development of effective and sustainable interventions for children and youth [82]. In the present intervention, the gender-sensitive concept was partly based on each participant’s own needs and suggested themes for discussion – consequently, these themes addressed complex *gendered* stressors and demands. We believe that this dynamic and participatory course pedagogy was a strength, which also encouraged participants to take part and be active in their own treatment and solutions.

### Methodological considerations

The project’s strong base within an already existing and well-established youth-friendly health service was a clear strength in the initial phase and in the implementation of the intervention. The building of trust was reinforced by the supportive and competent milieu at the centre, with experienced personnel and opportunities for the young women to receive professional support, if needed. The first author’s (MS) involvement (also as a the leader of the intervention) generated both potential weaknesses and strengths. Being both group leader and researcher may have led to less critical and negative views from the participants during the follow-up interviews. On the other hand, the close contact with participants and familiarity with the course content may have created a trusting atmosphere that led to rich accounts. We also believe that the first author’s closeness to participants and the centre was counterbalanced by the other authors’ more distanced positions. During the data collection and analysis, members of the research group thus had different, and fluctuating, roles in terms of having an ‘insider’ or an ‘outsider’ perspective – which we define as a strength. In addition, through the first author’s close involvement, the project was well grounded and could be realised within a natural and clinical setting. In the next step, this facilitated the implementation of the intervention in ordinary services after the completion of the project period.

Trustworthiness and credibility in the study were achieved through the researchers’ (MS, MW) prolonged engagement, and through triangulation among researchers and between empirical data and theory [83]. Triangulation between researchers was performed through our different clinical and theoretical competences and perspectives. Also, the gender-theoretical frame functioned as a critical lens that brought additional perspectives into the analysis and interpretation of the data. Quotations presented in the results further strengthened the trustworthiness of the study [61]. In terms of the ability to transfer and generalise results from the present study, we are well aware that the group studied may be considered as specific, because participants were motivated to apply for support and intervention in a group context; they were also homogeneous regarding ethnicity, for example. However, we still believe that results are possible to compare with, and eventually transfer to, other similar groups of young women or similar social contexts. On the whole, we believe that this intervention study contributes to valuable knowledge regarding the development of gender-sensitive and health-promoting interventions for both the target group and other groups of young people. We suggest, however, that future gender-sensitive interventions should include additional subgroups of girls and young women.

## Conclusions

Based on our analysis, it is clear that the group intervention worked to facilitate coping, action, and change in a hectic and demanding life. The participating young women’s experiences of social support from similar others, described as a social oasis, led to insights and new explanatory models where collective understanding and a shared gendered identity helped them to reduce the individual responsibility for, and feelings of failure and stigma associated with, distress and help seeking. The participants experienced the intervention as a safe and explorative space for gendered identity work and personal integrity as well as an explorative bodily space to discover embodied resources such as strength, stability, and relaxation. As such, the group also worked as a ‘breathing space’ in a context of multiple pressures. On a more tangible level, the body-based elements in the intervention model served to positively moderate and decrease stress-related symptoms such as anxiousness, restlessness, muscle tension, aches and pains, fatigue, and impaired sleep.

Within this safe space for gendered and embodied empowerment, the young women could challenge their social positions and stance towards life by upgrading and protecting themselves and their needs, changing pace, and setting limits. However, these findings also illustrate their personal burden of responsibility and their continued and still ongoing struggles and negotiations to handle internal and external pressures, such as complex gendered ideals and sociocultural expectations. Thus, we find there is a need to develop gender-sensitive group interventions to decrease this individualisation of health problems among youth, and instead encourage spaces for collective support, action, and change.

## Competing interests

The authors declare that they have no competing interests.

## Authors’ contributions

MW and EBMO were responsible for the overall project and study design. MS and MW planned the intervention, and MS led all the groups. MS and MW collected the data. MS prepared the data and was mainly responsible for the initial data analysis. All authors contributed to the interpretation of data. MS and MW drafted the manuscript. All authors critically revised, read, and approved the final manuscript.

## Authors’ information

Maria Strömbäck is a physiotherapist specialising in youth psychiatry, drug abuse, psychosomatics, basic body awareness therapy (BBAT) and cognitive behavioural counselling. She is a PhD student at the National Graduate School of Gender Studies and also affiliated with the Physiotherapy and Psychiatry units at Umeå University, Umeå, Sweden. Her PhD studies concern mental ill-health and the development of gender-sensitive group interventions geared towards young people within psychiatry and youth health services. She is a researcher in the project Stress and Health in Youth (Umeå SHY).

Eva-Britt Malmgren-Olsson, PhD in Physiotherapy, is a senior lecturer in Physiotherapy, Department of Community Medicine and Rehabilitation, Umeå University, Sweden. She is a senior researcher specialising in musculoskeletal pain with special reference to body awareness treatment and stress management. She is a researcher in the project Stress and Health in Youth (Umeå SHY).

Maria Wiklund, PhD in Public Health, holds a post-doc position at Umeå Centre for Gender Studies (UCGS) and is a lecturer in Physiotherapy, Department of Community Medicine and Rehabilitation, Umeå University, Sweden. She is also a physiotherapist specialising in psychosomatics, pain, stress, trauma, and basic body awareness therapy (BBAT). Her research focus is on stress, body, health, and the development of health-promoting gender-sensitive interventions among young people. She is the group leader of the research project Stress and Health in Youth (Umeå SHY).

## Pre-publication history

The pre-publication history for this paper can be accessed here:

http://www.biomedcentral.com/1471-2458/13/907/prepub
